# Particle-Size Distribution Models for the Conversion of Chinese Data to FAO/USDA System

**DOI:** 10.1155/2014/109310

**Published:** 2014-07-07

**Authors:** Wei Shangguan, YongJiu Dai, Carlos García-Gutiérrez, Hua Yuan

**Affiliations:** ^1^College of Global Change and Earth System Science, Beijing Normal University, No. 19, Xinjiekouwai Street, Beijing 100875, China; ^2^Department of Applied Mathematics, School of Agricultural Engineering Technical, University Polytechnic of Madrid, 28040 Madrid, Spain

## Abstract

We investigated eleven particle-size distribution (PSD) models to determine the appropriate models for describing the PSDs of 16349 Chinese soil samples. These data are based on three soil texture classification schemes, including one ISSS (International Society of Soil Science) scheme with four data points and two Katschinski's schemes with five and six data points, respectively. The adjusted coefficient of determination *r* ^2^, Akaike's information criterion (AIC), and geometric mean error ratio (GMER) were used to evaluate the model performance. The soil data were converted to the USDA (United States Department of Agriculture) standard using PSD models and the fractal concept. The performance of PSD models was affected by soil texture and classification of fraction schemes. The performance of PSD models also varied with clay content of soils. The Anderson, Fredlund, modified logistic growth, Skaggs, and Weilbull models were the best.

## 1. Introduction

Particle-size distribution (PSD) is a basic physical property of soils that affects many important soil properties. PSD has been widely used for estimating various soil hydraulic properties [1, 2]. The PSD prediction has been used for comparing and converting texture measurements from different classification systems [3–6]. Many soil databases do not contain detailed PSD data but contain only several mass fractions and use different cutoff points to separate the size fractions. To obtain a more complete description of texture, various parametric PSD models are used [7–12]. In the Second National Soil Surveys of China, soil textures were measured by ISSS (International Society of Soil Science) and Katschinski's systems. The conversion from ISSS and Katschinski's to the popular USDA (United States Department of Agriculture) system is required to achieve compatibility of soil data and to use pedotransfer functions as estimators of soil hydraulic properties [13, 14].

PSD models can be categorized as several classes: regression [15], spline [13], fractal method [16, 17], method based on PSD statistics [12], similarity procedure [13], gray model [18], and so on. In this study, we investigated regression models for their simplicity and effectiveness when there are a small number of fractions.

A few studies have been performed with the purpose of determining the best model for fitting particle-size distribution curves of soils [9, 15, 19–23]. Hwang et al. [15] compared seven PSD models to fit PSD data sets of Korean soils and found that the Fredlund model with four fitting parameters [11] showed the best performance for the majority of texture types. Hwang [19] examined nine PSD models and found that the performance of the PSD models was affected by soil texture and most of them improved with an increase in clay content. He also reported that the Fredlund model and two Skaggs models [12] showed better performance for several specific soil textures. Liu et al. [20] evaluated the suitability of five models to fit particle-size analysis data of Chinese soils of three texture types. The results indicated that the four-parameter Fredlund model showed the best representation of particle-size distributions, and the three-parameter Fredlund model and modified logistic growth model produced comparative results for silty clay loam and silt loam soils but yielded worse results for sandy loam soils. da Silva et al. [21] tested 14 different models with feasibility to fit the cumulative soil particle-size distribution curve based on four measured points and experienced that the most recommendable models were the Skaggs et al. [12], Lima and da Silva [24], Weibull [25], and Morgan et al. [26] models, which are all three-parameter models. Botula et al. [23] evaluated ten PSD models for soils of humid tropics and found that the performance was dependent on the bimodal character of the soil PSD. However, these studies have not fully investigated the performance of PSD models and used data measured only according to USDA standard.

Several studies have proposed conversion methods between texture classification systems. Nemes et al. [13] evaluated the accuracy of four interpolation procedures using a data set of European countries, and these procedures were used to accomplish the standardization of particle-size description by the FAO (Food and Agriculture Organization)/USDA system. Minasny and McBratney [4] used an empirical multiple linear regression to accomplish the conversion between the ISSS and USDA system. Shirazi et al. [27] developed a conversion table between the USDA and ISSS system based on a lognormal particle-size distribution assumption within each size fraction (sand, silt, and clay). Shirazi et al. [5] expanded the development of the PSD statistics (i.e., the geometric mean particle diameter and its standard deviation) to compare USDA and ISSS systems including rock fragments, which can be applied to the conversion between the two systems. Most PSD models are incapable of supporting the conversion from Katschinski's to USDA systems. These PSD models are not capable of extrapolating the soil fraction above the upper limit (i.e., 1 mm) of Katschinski's system, but the fraction between 2 mm and 1 mm is included by the USDA system. Few studies have been devoted to the conversion between USDA and Katschinski's systems [3]. The conventional method is plotting the particle-size curve by semilogarithm regression [28], but it is time-consuming and of low accuracy. Rousseva [3] used the fraction between 1 mm and 3 mm to interpolate the cumulative fraction at 2 mm limit by exponential and power-law distribution models during the transformation from Katchinski's to ISSS and USDA systems. However, the gravel contents are usually not measured (e.g., the Second National Soil Surveys of China). The following question arises: is it possible to extrapolate the soil fraction between 2 mm and 1 mm based on the data below 1 mm limit? The fractal method [29] provides an optional solution, which was used in this work.

The objective of this study is to evaluate the performances of several PSD models to find the best describing the soil PSD, to convert the data from ISSS and Katschinski's schemes to the USDA standard, and to investigate the effects of soil texture and classification schemes on the efficiency of PSD models.

## 2. Material and Methods

### 2.1. The Chinese Soil Profile Database

The Chinese soil profile database was established using the results of the Second National Soil Surveys of China conducted in the 1980s. It contains data from 33010 soil horizons representing 8979 profiles. The data have been published by the National Soil Survey Office [30], soil survey offices of provinces, and some Tibetan counties' soil survey offices. This work collected and digitized these data, which are available in Microsoft Excel worksheet file format. Not all soil horizons have PSD data. Fine size fractions were determined by the hydrometer or pipette method, whereas the coarse fractions were obtained by sieving [28]. Particle-size fraction data were classified by ISSS or Katschinski's schemes. Katschinski's [31] scheme was not only used in China but also widely used in countries of former USSR and Eastern Europe. Because some schemes have small number of soil samples and some schemes have too few particle-size limits (e.g., 2, 0.02, and 0.002) for PSD models to fit the curve, we used only part of the data (i.e., 16349 samples) in this study. Four to six particle-size fractions are given in one ISSS scheme and in two Katschinski's schemes (Table 1).

### 2.2. Particle-Size Distribution Models

Eleven PSD models were tested in this study (Table 2). Ten models are shown in Table 2 and a self-similar model (SELF) is described here because this model cannot be given in a simple equation [32] and it has not yet been compared with other models in previous studies.

The self-similar model uses the soil textural data to define an iterated function system, which determines how the self-similar distribution reproduces its fractal structure at different length scales [33]. A set of *N* proportions of soil mass are selected (for simplification, assume that *N* = 3). The fractal model for PSD derived from the self-similarity hypothesis is constructed as follows (see Martín and Taguas [32] for the details): let *I*_1_ = [0, *α*_1_], *I*_2_ = [*α*_1_, *α*_2_], and *I*_3_ = [*α*_2_, *c*] be the subfractions of sizes corresponding to the three size classes and let *p*_1_, *p*_2_, and *p*_3_ be the proportions of mass for the fractions *I*_1_, *I*_2_, and *I*_3_, respectively. Notice that *p*_*i*_ > 0 and *p*_1_ + *p*_2_ + *p*_3_ = 1. Associated with these definitions, we consider the following functions: *φ*_1_(*x*) = *r*_1_ · *x*, *φ*_2_(*x*) = *r*_2_ · *x* + *α*_1_, and *φ*_3_(*x*) = *r*_3_ · *x* + *α*_2_, where *r*_1_ = *α*_1_/*c*, *r*_2_ = (*α*_2_ − *α*_1_)/*c*, and *r*_3_ = (*c* − *α*_2_)/*c* and *x* is any point (or particle size value) in the fraction *I* = [0,  *c*]. *φ*_1_, *φ*_2_, and *φ*_3_ are the linear mappings (similarities) that transform the points of the fraction *I* in the points of the fractions *I*_1_, *I*_2_, and *I*_3_, respectively. The set {*φ*_*i*_, *p*_*i*_, *i* = 1, 2,3} is called an iterated function system (IFS) and it defines a self-similar distribution *μ* supported on [0, *c*], which satisfies *μ*(*I*_*i*_) = *p*_*i*_ for every *I*. The set of textural data, together with the self-similarity hypothesis mentioned above, determine a self-similar mass distribution *μ* for particle size determined in *I*, which fits exactly the input data; that is, *μ*(*I*_*i*_) = *p*_*i*_. From the self-similarity, the contribution to soil mass of particles with sizes in the fraction *I*_*ij*_ = *φ*_*j*_(*I*_*i*_) is given by *μ*(*I*_*ij*_) = *p*_*i*_ *p*_*j*_. In general, for any size fraction *J* ⊂ *I*, the IFS model allows one to determine the mass of soil *μ*(*J*) corresponding to the soil particles with sizes in the fraction *J*. In this study, the values of the fraction limits chosen are *α*_1_ = 10, *α*_2_ = 100, and *c* = 1000 (in *μ*m); and the model is seen as a biparametric model (*p*_1_ and *p*_2_) which varies until the best simulation, in terms of minimizing the distance between simulated and real data, is reached.

Six models which showed relatively good performance in the work of Hwang et al. [15, 19] were tested, including the Fredlund4P (F4P), Skaggs (S), van Genuchten (VG), Weibull (W), offset-nonrenormalized lognormal (ONL), and offset-renormalized lognormal (ORL) models. In addition, four other models with different number of fitting parameters were tested. These models are selected among scores of PSD models in the literature according to their performance in previous studies. The Anderson model (AD) [8] assumes that the log mass of the particles is arcus-tangent distributed (i.e., Cauchy distributed). Fredlund et al. [11] found that a value of 0.001 for the parameter *d*_*f*_ in F4P model (Table 2) provided a reasonable fit in most cases, so the Fredlund3P (F3P) model with three parameters was also tested. The modified logistic growth model (ML) [20] is modified from the logistic growth model whose graph is in a sigmoid shape. The van Genuchten type model is modified by Zhuang et al. [34] (VGM) which is also based on an assumption that the shape of particle-size distribution is sigmoidal. Spline is not included because Liu et al. [20] found that it performs worse than modified logistic model.

### 2.3. Model Comparison and Fitting Techniques

We used three statistical indices to determine the performance of the PSD models. The adjusted coefficient of determination (*r*_adj_^2^) was used as a relative measure of goodness-of-fit between predicted data and observed data, which is a better criterion than the coefficient of determination [23]. *r*_adj_^2^ is defined as

(1) $$\begin{array}{c} r_{\text{adj}}^{2}=1-\frac{N-1}{N-P}\left(1-r^{2}\right) \end{array}$$

with

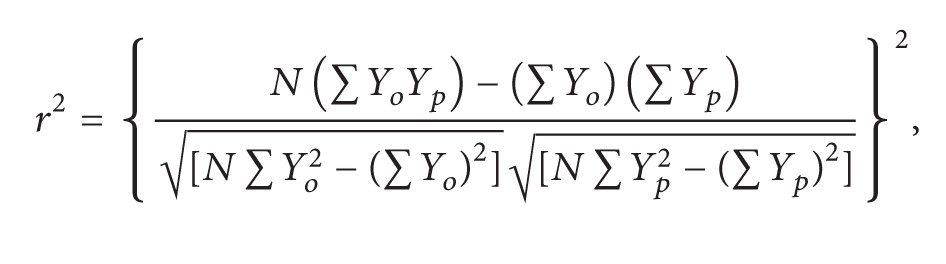
(2)
where *N* is the number of observed data points, *P* is the number of model parameters, and *Y*_*o*_ and *Y*_*p*_ are the observed and predicted cumulative mass fractions, respectively. A further index was Akaike's information criterion (AIC) [35] which examines the complexity of the model together with its goodness of fit to the sample data, balances between the two, and discourages overfitting. AIC is defined as

(3) $$\begin{array}{c} \text{AIC}=N\ln\left(\text{RSS}\right)+2P \end{array}$$

with

(4) $$\begin{array}{c} \text{RSS}=\sum {\left(Y_{o}-Y_{p}\right)}^{2}, \end{array}$$

where RSS is the residual sum of squares. Geometric mean error ratio (GMER) was used to test whether the underestimation (values bigger than 1) or overestimation (values smaller than 1) in model's prediction occurs:

(5) $$\begin{array}{c} \text{GMER}=\exp\left(\frac{1}{N}\sum \ln\left(\frac{Y_{p}}{Y_{o}}\right)\right). \end{array}$$

The “soiltexture” package of *R* language was developed to fit the parametric models to the raw data [36]. The optimization methods in the “optim” function of *R* are used, including Nelder-Mead, quasi-Newton and conjugate-gradient, box-constrained, and simulated annealing algorithms [37]. We ran all methods and chose the best results with minimum RSS. The nonlinear optimization procedures were carried out using at least five random initial parameter estimates for all soils. When the final solution for each soil converged to different parameter values, the parameter values with the best fitting statistics (i.e., RSS) were kept. In most cases, the parameters were similar.

### 2.4. Conversion to the USDA Standard

The soil data were converted to the USDA standard, including soil fractions (i.e., clay fraction (*d* < 0.002 mm), silt fraction (0.002 < *d* < 0.05 mm), and sand fraction (0.05 < *d* < 2 mm)).

After estimating the parameters of each model for every soil sample, we can easily obtain the predicted values of the cumulative mass fractions at a specific point between the upper and lower limits of the three soil fraction schemes, so the fractions in USDA standard can be obtained. The result of the model with the biggest *r*_adj_^2^ value was adopted for a sample in the conversion to the USDA standard. For T1 scheme, the clay fraction was already known, the silt fraction was the predicted cumulative fraction at the observed 0.05 mm limit minus clay fraction, and the sand fraction was calculated by subtracting silt fraction and clay fraction from 100%. For T2 and T3 scheme, the clay fraction was predicted by PSD models, and the silt fraction was calculated by subtracting clay fraction from the cumulative fraction at the observed 0.05 mm limit. To predict the cumulative mass fraction at the 2 mm limit, the fractal method was adopted. In recent years, PSD of soil samples has been successfully studied by fractal methods. Turcotte [16] and Tyler and Wheatcraft [17] developed a relationship relating mass measurements and diameters for the analysis of PSD, which is expressed as

(6) $$\begin{array}{c} F\left(R\right)=\frac{M\left(r<R\right)}{M_{T}}={\left(\frac{R}{R_{L}}\right)}^{3-D}, \end{array}$$

where *M*(*r* < *R*) is the mass of soil particles with a radius smaller than a prespecified size *R*; *M*_*T*_ is the total mass of particles; *R*_*L*_ is the upper particle size limit; and *D* is the fractal dimension of fragmentation. The fractal dimension of fragmentation can be found by estimating the slope coefficient of the log⁡[*M*(*r* < *R*)/*M*_*T*_] versus log⁡[*R*] plot. Many studies using detailed experimental data have shown multiple fractal dimensions on log-transformed PSD of soil samples. Wu et al. [38] found three domains within PSDs determined over six orders of magnitude in the particle size. Bittelli et al. [39] reported that three main power-law domains could characterize the PSD across the whole range of measurements, including the sand domain between 0.0853 ± 0.0253 and 2 mm. Millán et al. [40] divided the whole PSD domain into two fractal domains (i.e., 0 < *d* < 0.2 mm and 0.2 < *d* < 2 mm), where the 0.2 < *d* < 2 mm domain spanned approximately one order of magnitude with fractal concepts. Prosperini and Perugini [41, 42] found that all soils in their analysis displayed two scaling domains in the range from 0.0014 to 50.8 mm. According to these studies, the slope of the log-log plot between the cumulative fractions and particle sizes has a linear trend in the domain from 0.5 to 2 mm. The relationship between the cumulative fraction at 2 mm and the cumulative fractions at 1 and 0.5 mm can be derived from (6). The equation is given by

(7) $$\begin{array}{c} F\left(2\right)=10^{2\log(F(1))-\log(F(0.5))}, \end{array}$$

where *F*(2), *F*(1), and *F*(0.5) are cumulative fractions with a diameter smaller than 2, 1, and 0.5 mm, respectively. *F*(1) and *F*(0.5) were predicted by PSD models. All three fractions were divided by the predicted value at 2 mm so that the cumulative final value was 100 percent. We did not apply the fractal model alone to do the conversion, because there are not enough observed data points to determine the subdomains and fractal dimensions to accomplish the interpolation for each soil sample in this study.

## 3. Results and Discussion

### 3.1. Model Performance

**Figure 1:**
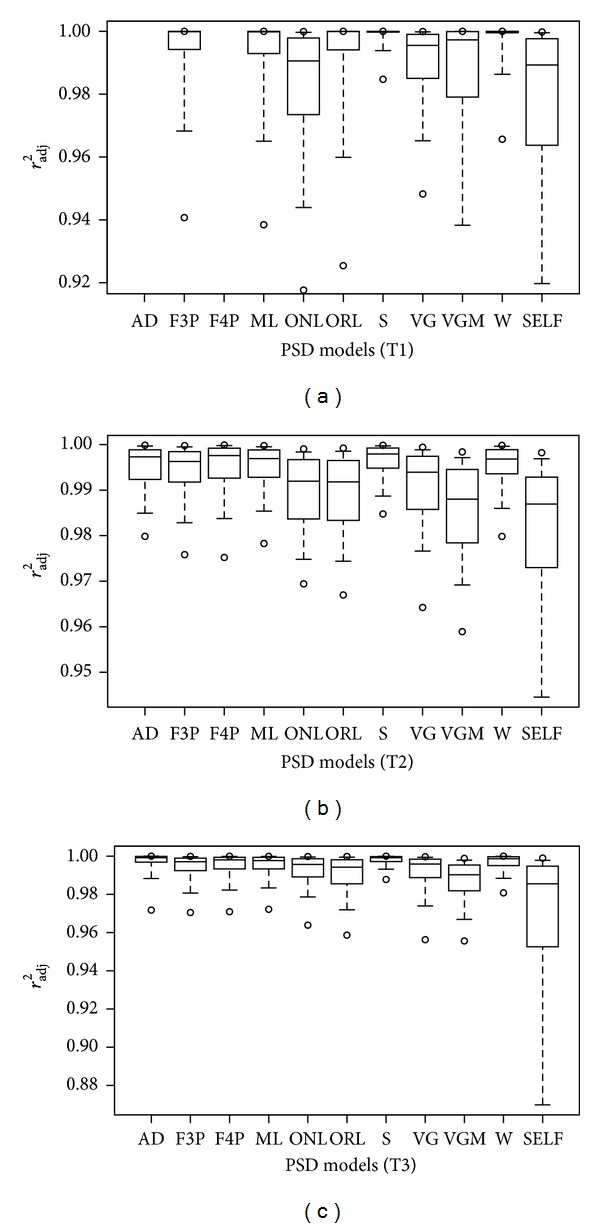
Box plot for *r*_adj_^2^ percentiles as the goodness-to-fit of 11 models, (a) T1 scheme soils, (b) T2 scheme soils, and (c) T3 scheme soils. AD = Anderson model, F3P = Fredlund3P model, F4P = Fredlund4P model, ML = modified logistic growth model, ONL = offset-nonrenormalized lognormal model, ORL = offset-renormalized lognormal model, S = Skaggs model, VG = van Genuchten type model, VGM = van Genuchten type modified model, W = Weibull model, and SELF = self-similar model. There is no box plot for AD and F4P model because *r*_adj_^2^ cannot be calculated for them.

**Figure 2:**
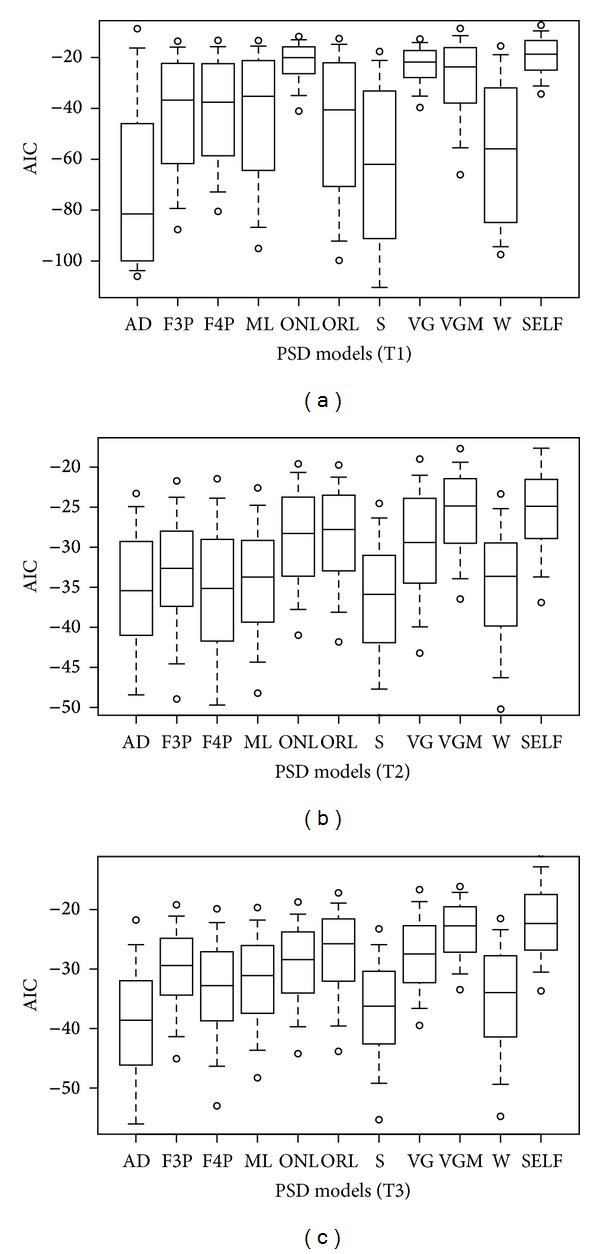
Box plot for Akaike's information criterion (AIC) percentiles as a criterion for assessing the performance of 11 models, (a) T1 scheme soils, (b) T2 scheme soils, and (c) T3 scheme soils.

Among all of the three soil fraction schemes and all of the models, values of *r*_adj_^2^ ranged from 0.873 to 1 (Figure 1). In T1 scheme of ISSS, the S and W models showed the highest *r*_adj_^2^ values; F3P, ML, and ORL models gave similar performance; and the other four models (ONL, VG, VGM, and SELF) had lower *r*_adj_^2^ values. In the two Katschinski's schemes (i.e., T2 and T3), the performances of PSD models were quite similar to each other. The S model with two parameters yielded the highest *r*_adj_^2^ values. The AD, F3P, F4P, ML, and W models indicated similar performance. The other five models had lower *r*_adj_^2^ values. The F4P model with four parameters performed slightly better than the F3P model with three parameters in both Katschinski's schemes, which indicates that F3P is enough for most soils with the advantage of one less parameter [11]. The ONL model had lower *r*_adj_^2^ values in the T1 scheme of ISSS than in Katschinski's scheme, while the ORL had better performance in the T1 scheme. The performance of AD model became better from the T2 scheme to the T3 scheme as one limit (0.25 mm) of PSD is omitted in T3, while the opposite happened to the SELF model. The results of the AIC test were generally consistent with those of *r*_adj_^2^ assessment (Figures 1 and 2). The performance of AD and F4P model with four parameters in the T1 scheme can be evaluated by AIC but not by *r*_adj_^2^, because the denominator (i.e., *N*-*P*) will be zero in (3). The AD model had the lowest AIC, while the F4P had similar AIC with the F3P model. Though AIC has penalty on parameter number, the AD model with four parameters and the W model with three parameters were still among the best PSD models.

**Figure 3:**
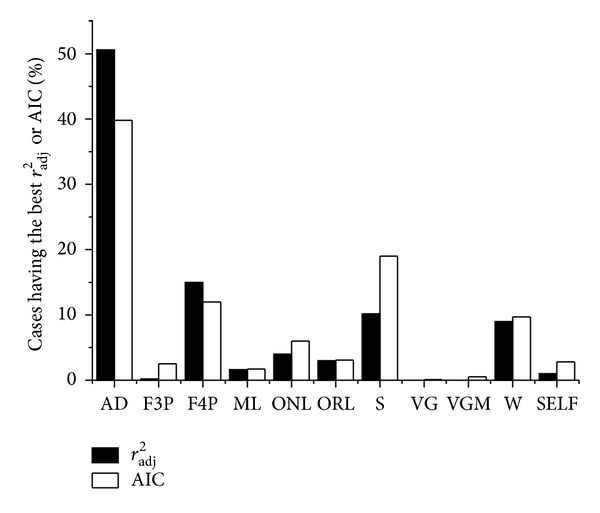
Percentage of soils of T3 scheme for which *r*_adj_^2^ or AIC are the best for a given model.

Though the performances evaluated by *r*_adj_^2^ and AIC were quite similar on the whole (Figures 1 and 2), the best model according to different criteria can be different for a specific soil.  Figure 3 shows the percentage of cases where a model was the best according to *r*_adj_^2^ and AIC for soils of the T3 scheme. The percentage of best cases according to *r*_adj_^2^ for the AD and F4P models was smaller than that according to AIC, while the opposite happened to the S and ONL models.

**Figure 4:**
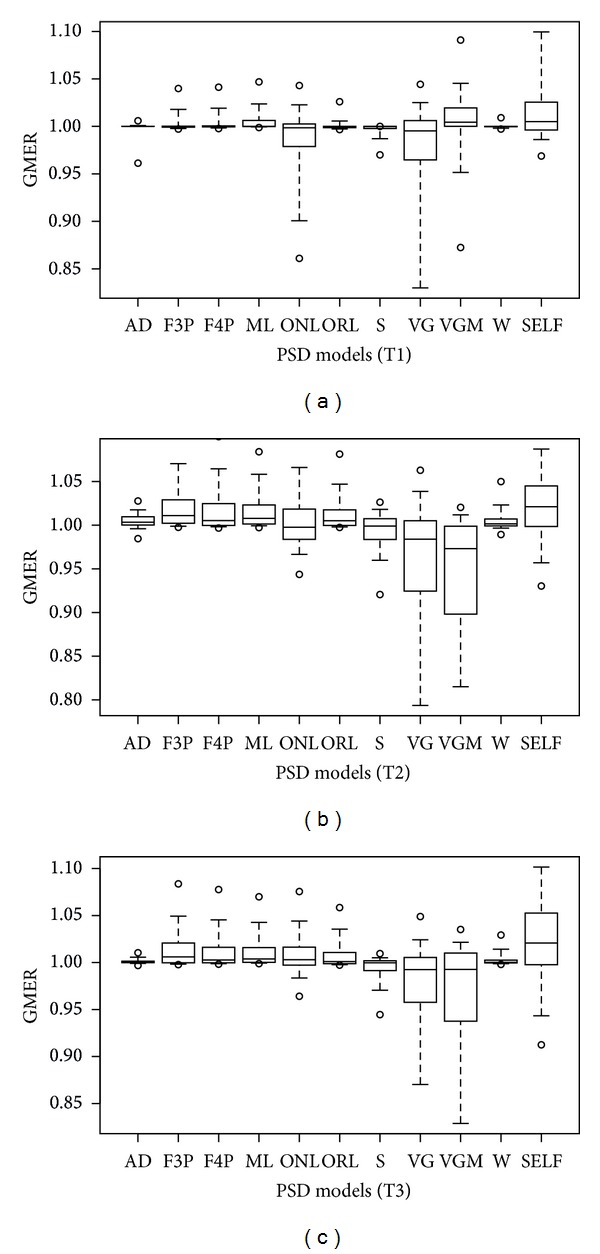
Box plot for geometric mean error ratio (GMER) percentiles as a criterion for assessing the performance of 11 models, (a) T1 scheme soils, (b) T2 scheme soils, and (c) T3 scheme soils.

Figure 4 shows GMER for all of the three soil fraction schemes and all of the models. Most models overestimated the fractions while the S and VG model underestimated them in general. The VGM model showed an overestimation in T1 scheme but it showed an underestimation in T2 and T3, while the opposite happened to the ONL model. The AD and W models showed the best results without apparent underestimation or overestimation.

Over all, the AD, S, and W models provided the best performance for soils in ISSS and Katschinski's schemes according to the *r*_adj_^2^ analysis, AIC tests, and GMER. The F4P, F3P, and ML models also displayed relative good performances.

### 3.2. The Validation of Combined Use of PSD Model and Fractal Method

**Figure 5:**
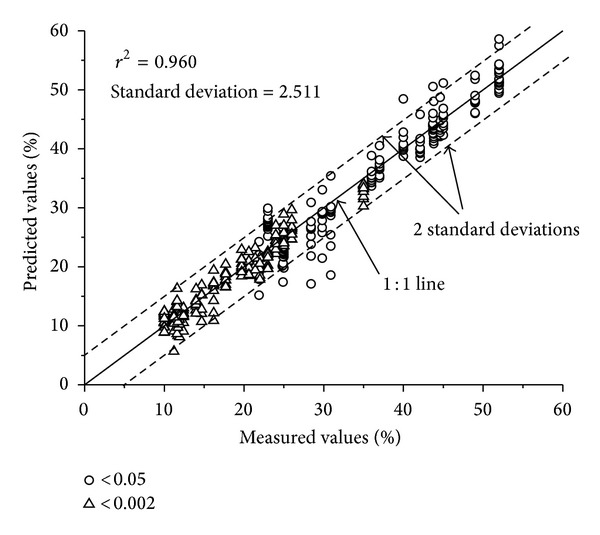
Percentages predicted by one of the eleven PSD models and fractal method versus measured percentages of particles finer than 0.05 mm and 0.002 mm of 20 soils.

PSD models to interpolate and fractal method to extrapolate have different statistical assumptions. We need to know whether the combined use of two distinct models results in a real representing soil sample. For this, 20 soil samples were used to validate the combined use, which has the observed cumulative particle-size percentages at 2 mm, 0.05 mm, and 0.002 mm of USDA standard and at the limits of T2 scheme, respectively. These samples were only available with the given specifics. We used each PSD model combined with the fractal method to predict PSD with data points of T2 scheme.  Figure 5 illustrates the test of suitability of the combined method to predict percentages of particles finer than 0.05 and 0.002 mm. The *r* ^2^ value was 0.960, which was slightly lower than those of PSD models. Thus, the combined method with interpolation and extrapolation is suitable for transferring data from Katschinski's to USDA scheme. However, it cannot be determined whether this method is suitable for a specific soil textural class, because the sample size is too small.

### 3.3. Effect of Soil Textural Classes and Fraction Schemes

The AIC analysis had revealed that the performance of a PSD model can be affected by soil textural classes and soil fraction schemes (Tables 3 and 4). The dataset used in this study represents all the 12 textural classes of ISSS system and 10 textural classes of Katschinski system but in various degrees (Tables 3 and 4). The dominant textural classes in the ISSS scheme are light clay (25.7%), clay loam (21.7%), sandy loam (15%), and loam (9%). The dominant textural classes in the T2 scheme are light clay (40.4%), moderate clay (27.6%), heavy loam (14.8%), and heavy clay (12%). The dominant textural classes in the T3 scheme are light clay (33.6%), moderate clay (21%), heavy loam (19%), and moderate loam (11.3%).  Table 3 shows the number of cases as the best model due to the smallest AIC value for each soil textural class of ISSS in the T1 scheme. All the models performed best in some cases. The AD model had the largest number of cases for most texture, while the S model did so for sand and silt clay. The AD model had the largest number of cases as the best model, followed by the S model. Although the S and W models performed similarly in the *r*_adj_^2^ and AIC analysis (Figures 1 and 2), the S model performed better with a bigger number of cases which resulted in the smallest AIC values for all soil textural classes. Although the SELF model did not perform well in the overall analysis (Figures 1 and 2), it had similar number of best cases with the W model. These results suggest that the PSD models that show good performance for overall soil PSDs are not always good models for each soil textural class.  Table 4 shows the number of cases of all the tested PSD models having the smallest AIC value for each soil textural class in the T2 and T3 schemes. In the T2 scheme (Table 4(a)), most soils are clay soils in Katschinski system (i.e., heavy clay, moderate clay, and light clay). The AD model had the largest number of cases which resulted in the smallest AIC values for these clay soils. However, the S model performed better for heavy loam. In the T3 scheme (Table 4(b)), the AD model also had the largest number of cases having the smallest AIC values for almost all soil textures, though the F4P, S, and W models also have a large number of cases. From the comparisons among the T1, T2, and T3 schemes, it appears that the performance of PSD models vary not only with the soil textural classes but also with the soil fraction schemes. However, the dataset used in this work does not seem to be sufficient to draw a well-founded conclusion for some textural classes with a small number of samples, especially in the T2 and T3 schemes.

**Figure 6:**
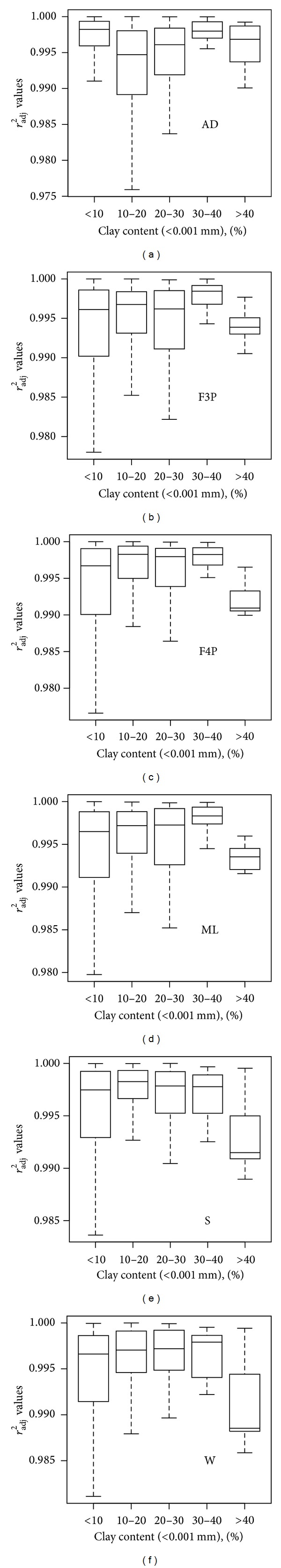
Box plot for *r*_adj_^2^ percentiles of the PSD models on different clay content of soils in the T2 scheme.

The performance of PSD models appears to be affected by clay content of soils in different manners. In the T2 scheme (Figure 6), the clay content is defined as particles smaller than 0.001 mm in Katschinski system. All models performed best with the clay content between 30% and 40%; the AD model had the smallest *r*_adj_^2^ values with clay content between 10% and 20%, and other models had the smallest *r*_adj_^2^ values with clay content larger than 40%; the S and W model became better with the increase in the clay content but were very poor with the clay content over 40%. However, the performance of these models with different clay content in the T1 and T3 schemes is quite different from that in the T2 scheme (results not shown here). Hwang et al. [15] found better fitting with the increase in the clay content. Botula et al. [23] observed no clear trend of performance with clay content using a more clayed soil database. Our results generally confirmed the findings of Botula et al. [23].

## 4. Conclusions

Eleven models for soil PSD were compared, including the AD, F4P, F3P, ML, ONL, ORL, S, SELF, VG, VGM, W, and SELF models, using one ISSS scheme (i.e., T1) data with four data points and two Katschinski's schemes (i.e., T2 and T3) with five and six data points. The results from *r*_adj_^2^ values, AIC, and GMER test indicated that the AD, F4P, F3P, S, and W models had the best performance in three schemes. The combined use of PSD model and fractal method was suitable for transferring soil texture data from Katschinski's to USDA scheme when there are only sparse observed data points. Soil texture can affect the performance of PSD models. And the performance of PSD models has some difference using experimental data of different soil fraction schemes. The performance of PSD models can be affected by clay content of soils in quite different manners for different classification of soil fraction schemes. This work contributes to the selection of soil PSD models to describe the soil particle-size distribution curve which can be used to estimate the hydraulic properties and to compare and convert the different soil texture classification systems.

## Figures and Tables

**Table 1:** Soil particle-size data used in this study.

Soil fractions schemes	The particle-size limits (mm)	Number
T1 (ISSS^†^)	2, 0.2, 0.02, 0.002	15262
T2 (Katschinski^‡^)	1, 0.25, 0.05, 0.01, 0.005, 0.001	671
T3^§^ (Katschinski)	1, 0.05, 0.01, 0.005, 0.001	1090

^†^ISSS: international system of International Soil Science Society.

^‡^Katschinski: Katschinski classification system of Russia.

^§^T3 contains the data of T2 after summing up the 1–0.25 mm and 0.25–0.05 mm fraction.

**Table 2:** Particle-size distribution models.

Name	Model^†^	Parameters
Anderson (AD [8])	$F(d)=f_{0}+b\arctan\left(c\log\frac{d}{d_{0}}\right)$	*b, c, f*_0_, *d*_0_

Fredlund4P (F4P [11])	$F(d)=\frac{1}{{\left\{\ln\left[\exp(1)+{\left(a/d\right)}^{n}\right]\right\}}^{m}}\left\{1-{\left[\frac{\ln\left(1+d_{f}/d\right)}{\ln\left(1+d_{f}/d_{m}\right)}\right]}^{7}\right\}$	*a*, *n*, *m*, *d*_*f*_ (*d*_*m*_ = 0.0001 mm)

Fredlund3P (F3P [11])	$F(d)=\frac{1}{{\left\{\ln\left[\exp(1)+{\left(a/d\right)}^{n}\right]\right\}}^{m}}\left\{1-{\left[\frac{\ln\left(1+0.001/d\right)}{\ln\left(1+0.001/d_{m}\right)}\right]}^{7}\right\}$	*a*, *n*, *m* (*d*_*m*_ = 0.0001 mm)

Modified logistic growth (ML [43])	$F(d)=\frac{1}{\left[1+a\exp\left(-bd^{c}\right)\right]}$	*a*, *b*, *c *

Offset-nonrenormalized lognormal (ONL [9])	*G*(*X*) = *F*(*X*) + *c*, where *X* = In⁡(*d*) ${F(X)=\frac{{1+\mathrm{erf}\left[\left(X-\mu )/(\sigma \sqrt{2}\right)\right]}^{‡}}{2\left(X\geq \mu \right)}}^{}$ $F(X)=\frac{1-\mathrm{erf}\left[\left(X-\mu )/(\sigma \sqrt{2}\right)\right]}{2\left(X<\mu \right)}\;$	*μ*, *σ*, *c*

Offset-renormalized lognormal (ORL [9])	*G*(*X*) = (1 − ε)*F*(*X*) + ε [*F*(*X*) defined in ONL model]	*μ*, *σ*, *ε*

Skaggs (S [12])	$F(d)=\;{\left\{1+\left(\frac{1}{F\left(d_{0}\right)}-1\right)\exp\left[-uD^{c}\right]\right\}}^{-1}$, where *D* = (*d* − *d*_0_)/*d*_0_ *d*_0_ = 0.002 mm for T1; *d*_0_ = 0.001 mm for T2, T3; *F*(*d*_0_): fraction < *d*_0_	*u*, *c *

van Genuchten type (VG [7])	*F*(*d*) = [1+(*d*_*g*_/*d*)^*n*^] ^−*m*^, where *m* = 1 − 1/*n*	*d*_*g*_, *n *

van Genuchten type modified (VGM [34])	*F*(*d*) = 1 − (1 − *F*_min⁡_)[1+(*ad*)^*n*^]^−*m*^, where *m* = 1 − 1/*n*; *F*_min⁡_, fraction of minimum particle size	*a*, *n *

Weibull (W [10])	*F*(*d*) = *c* + (1 − *c*){1 − exp⁡(−*aD* ^*b*^)}, where *D* = (*d* − *d*_min⁡_)/(*d*_max⁡_ − *d*_min⁡_) *d*_max⁡_ = 2 mm, *d*_min⁡_ = 0.002 mm for T1; *d*_max⁡_ = 1 mm, *d*_min⁡_ = 0.001 mm for T2, T3	*a*, *b*, *c*,

^†^ *d*: particle diameter in mm.

^‡^ *erf*⁡[]: error function.

**Table 3:** Number of cases as the best model with the smallest AIC value for each soil textural class in T1 schemes^†^.

Texture^‡^	AD	F3P	F4P	ML	ONL	ORL	S	VG	VGM	W	SELF	Sum
Sa	64	33	17	9	0	32	73^§^	0	0	45	47	320
LoSa	163^§^	39	53	46	3	37	153	0	0	78	54	626
Lo	799^§^	16	3	24	1	70	380	0	1	36	47	1377
SaLo	979^§^	56	55	36	22	35	589	0	4	189	312	2277
SaClLo	464^§^	25	56	23	7	12	229	0	2	31	97	946
SiClLo	244^§^	22	1	6	0	14	196	1	0	27	0	511
SiLo	137^§^	9	0	4	0	17	95	0	0	24	0	286
ClLo	1929^§^	31	0	62	9	72	1024	0	2	80	110	3319
SaCl	108^§^	4	16	3	0	1	36	0	0	7	6	181
SiCl	236	16	1	55	2	18	264^§^	3	0	39	0	634
LCl	2086^§^	33	34	138	1	66	1346	0	1	97	115	3917
HCl	385^§^	10	21	39	1	23	335	0	0	47	7	868

Sum	7594^§^	294	257	445	46	397	4720	4	10	700	795	15262

^†^For example, if the best model according to AIC value of a soil sample is the AD model, the number of the AD model in the table will increase by one. If a model has a big number in a soil textural class, it has advantages in fitting the PSD curve compared to models with small number. The compared eleven models are AD = Anderson model, F3P = Fredlund3P model, F4P = Fredlund4P model, ML = modified logistic growth model, ONL = offset-nonrenormalized lognormal model, ORL = offset-renormalized lognormal model, S = Skaggs model, VG = van Genuchten type model, VGM = van Genuchten type modified model, W = Weibull model, and SELF = self-similar model.

^‡^Textural classes of ISSS system: Sa = sand, LoSa = loamy sand, Lo = loam, SaLo = sandy loam, SaClLo = sandy clay loam, SiClLo = silty clay loam, SiLo = silt loam, ClLo = clay loam, SaCl = sandy clay, SiCl = silty clay, LCl = light clay, and HCl = heavy clay.

^§^The biggest number of cases as the best model for the specific soil texture class.

**(a):** 

Texture^†^	AD	F3P	F4P	ML	ONL	ORL	S	VG	VGM	W	SELF	Sum
SL	1^§^	1^§^	0	0	0	0	0	0	0	0	0	2
HL	17	5	7	4	8	2	32^§^	5	5	14	0	99
ML	8^§^	4	1	4	1	6	3	0	0	1	0	28
LL	2^§^	1	0	0	0	1	1	1	0	0	0	6
HC	38^§^	6	19	1	1	0	4	0	0	3	8	80
MC	62^§^	8	50	5	9	1	31	3	1	10	5	185
LC	79^§^	0	60	7	12	2	67	8	6	29	1	271

Sum	207^§^	25	137	21	31	12	138	17	12	57	14	671

**(b):** 

Texture^‡^	AD	F3P	F4P	ML	ONL	ORL	S	VG	VGM	W	SELF	Sum
TS	2^§^	1	1	0	0	0	0	0	0	0	0	4
LS	0	1	2^§^	0	0	0	0	0	0	0	0	3
SL	9^§^	2	1	0	1	2	5	0	0	0	0	20
HL	74^§^	1	16	10	22	7	45	0	3	23	6	207
ML	53^§^	7	9	4	6	15	14	0	1	13	1	123
LL	21^§^	4	7	1	0	5	4	1	0	4	0	47
HC	40^§^	7	14	0	0	0	12	0	0	7	11	91
MC	90^§^	1	37	2	9	7	44	0	0	30	9	229
LC	143^§^	5	39	0	39	3	85	1	4	39	8	366

Sum	432^§^	29	126	17	77	39	209	2	8	116	35	1090

^†^For example, if the best model according to AIC value of a soil sample is the AD model, the number of the AD model in the table will increase by one. If a model has a big number in a soil textural class, it has advantages in fitting the PSD curve compared to models with small number.

^‡^Textural classes of Katschinski system: TS = tight sand, LS = loose sand, SL = sandy loam, HL = heavy loam, ML = moderate loam, LL = light loam, HC = heavy clay, MC = moderate clay, and LC = light clay.

^§^The biggest number of cases as the best model for the specific soil texture class.
